# Comparing cognitive behavioural therapy for eating disorders integrated with behavioural weight loss therapy to cognitive behavioural therapy-enhanced alone in overweight or obese people with bulimia nervosa or binge eating disorder: study protocol for a randomised controlled trial

**DOI:** 10.1186/s13063-015-1079-1

**Published:** 2015-12-18

**Authors:** Marly Amorim Palavras, Phillipa Hay, Stephen Touyz, Amanda Sainsbury, Felipe da Luz, Jessica Swinbourne, Nara Mendes Estella, Angélica Claudino

**Affiliations:** Eating Disorders Programme (PROATA), Department of Psychiatry, Federal University of São Paulo (UNIFESP), São Paulo, Brazil; CAPES Foundation, Ministry of Education, São Paulo, Brazil; School of Medicine, Western Sydney University, Sydney, NSW Australia; Centre for Health Research, School of Medicine, Western Sydney University, Sydney, NSW Australia; School of Psychology, Faculty of Science, The University of Sydney, Camperdown, NSW Australia; The Boden Institute of Obesity, Nutrition, Exercise and Eating Disorders, Sydney, NSW Australia; Medical School, and School of Psychology, The Faculty of Science, The University of Sydney, Camperdown, NSW Australia

**Keywords:** Obesity, Binge eating, Psychotherapy, Weight management

## Abstract

**Background:**

Around 40 % of individuals with eating disorders of recurrent binge eating, namely bulimia nervosa and binge eating disorder, are obese. In contrast to binge eating disorder, currently there is no evidence base for weight management or weight loss psychological therapies in the treatment of bulimia nervosa despite their efficacy in binge eating disorder. Thus, a manualised therapy called HAPIFED (Healthy APproach to weIght management and Food in Eating Disorders) has been developed. HAPIFED integrates the leading evidence-based psychological therapies, cognitive behavioural therapy-enhanced (CBT-E) and behavioural weight loss treatment (BWLT) for binge eating disorder and obesity respectively. The aim of the present study is to detail the protocol for a randomised controlled trial (RCT) of HAPIFED versus CBT-E for people with bulimia nervosa and binge eating disorder who are overweight/obese.

**Method/Design:**

A single-blind superiority RCT is proposed. One hundred Brazilian participants aged ≥ 18 years, with a diagnosis of bulimia nervosa or binge eating disorder, BMI > 27 to < 40 kg/m^2^, will be recruited from both community and clinics and individually randomised to a therapy arm. Five groups of ten participants will receive the experimental intervention (HAPIFED) and the other five groups of ten the control intervention (CBT-E). Both therapies are manualised, and in this RCT will comprise 1 individual session and 29 office-based group sessions over 6 months. Assessment points will be at baseline, end of therapy, and 6 and 12 months after end of therapy. The primary outcome of this intervention will be reduced weight. Secondary outcomes will be improved metabolic indicators of weight management, reduction in eating disorder symptoms including improved control over eating, improved adaptive function, physical and mental health-related quality of life, and reduced levels of depression and anxiety.

**Discussion:**

This study will be the first to investigate a psychological therapy that aims to assist weight management in people with co-morbid overweight or obesity bulimia nervosa as well as with binge eating disorder. It will have the potential to improve health outcomes for the rapidly increasing number of adults with co-morbid obesity and binge eating disorder or bulimia nervosa.

**Trial registration:**

US National Institutes of Health clinical trial registration number NCT02464345, date of registration 1 June 2015.

**Electronic supplementary material:**

The online version of this article (doi:10.1186/s13063-015-1079-1) contains supplementary material, which is available to authorized users.

## Background

The combined general population lifetime prevalence of bulimia nervosa (BN) and binge eating disorder (BED) is around 5 % in women and 3 % in men [1]. Both disorders are characterised by recurrent binge eating or uncontrolled overeating episodes which, in the case of BN are also associated with regular compensatory weight control behaviours (e.g., self-induced vomiting) [2, 3]. It is now recognised that an increasing number, now around 40 % of people in the community with BN or with BED are obese [4, 5]. In clinics the prevalence is even higher, up to 80 % [6, 7]. Bulik et al. suggested, in a paper to highlight the issues of managing weight and eating disorders, that ‘BN in the overweight and obese patients may represent the natural evolution of the eating disorder on the backdrop of the obesity epidemic’ (Bulik et al., p.6) [8]. They wrote about the increase in clinical presentations of overweight and obese patients with BN and their requests for help with weight management. They argued that due to the rise in combined prevalence of co-morbid obesity and BN and BED, and their shared risk and maintaining features, an urgent need for new approaches to management is needed [8].

Patients with co-morbid BN or BED and obesity are currently faced with a problematic choice. Community-based studies [9, 10] indicate that while individuals with BN and BED frequently seek help in order to lose weight, most evidence-based psychological treatments such as cognitive behavioural therapy (CBT) for eating disorders fail to aid weight loss [11, 12]. In one study, Brody et al. [13] found the majority (63 %) of people with BED and co-morbid obesity chose CBT over a behavioural weight loss treatment (BWLT). However, 50 % of those who chose BWLT also thought their primary problem was BED and not obesity. Individuals with BN or BED and obesity may consequently undergo years of treatment for weight loss without attention to binge eating and its associated psychological co-morbidities [14, 15]. Further, whilst approaches such as BWLT for obesity are associated with weight loss in BED, weight regain is common [8] and whereas dietary restriction in normal weight people, with BN risks exacerbating the cycle of bingeing and fasting, as suggested by the classic Keys study of starvation [16], this may well differ for dietary restriction that is part of a supervised BWLT programme in people who are obese. As Bulik discussed, there may actually be harm from not addressing unchecked obesity [8]. A recent systematic review suggests that even very high levels of dietary restriction, as in low or very low energy diets, do not exacerbate binge eating, at least in BED [17]. Individuals with binge eating and obesity seek help to lose weight, but further research to establish the safety of weight loss treatment in BN is needed. In sum, BWLT fail to address the eating disorder and conversely psychological treatments fail to aid weight loss.

To address the need for treatment of BN and BED co-morbid with obesity, a manualised therapy called a Healthy APproach to weIght management and Food in Eating Disorders (HAPIFED) was developed (da Luz et al., manuscript in preparation). This therapy aims to integrate features of the leading evidence-based psychological therapies for BN and BED, CBT-enhanced (CBT-E) [18, 19] with a specific BWLT for weight loss and reduced binge eating in BED [20, 21]. In HAPIFED, like BWLT, dietary restriction and weight loss goals are modest (around 5 % of percent body weight) and a healthy lifestyle is promoted together with attention to the role of nutrition, food and eating in mood regulation. As recommended by Bulik et al. and Sainsbury-Salis [8, 22], internal appetite regulation and self-awareness of hunger and satiety cues are favoured over external regulation of food intake, and approaches are also informed by an understanding of effects of energy restriction [22]. HAPIFED differs from CBT-E in including obesity risk and maintaining factors in the personalised formulation, psychoeducation for obesity, self-monitoring of appetite regulation, weight loss management via behavioural activation and promotion of physical activity with the participation of a multidisciplinary team (psychologist, dietitian, physician and occupational or activity therapist). An important feature of HAPIFED is also the increased number of sessions (30), and the treatment period of 6 months, which is one third more sessions and 2 months longer than usual CBT-E [18].

Table 1 shows the similarities and differences between CBT-E and HAPIFED. To avoid bias from effects of therapy duration in this proposed randomised controlled trial (RCT), the CBT-E broad version will be used, which may be extended to 30 sessions, and has additional modules addressing psychological- maintaining factors, namely clinical perfectionism, core low self-esteem and interpersonal problems [18].

**Table 1:** Similarities and differences between the trial therapies

Included	HAPIFED	CBT-E
Personalised ED CBT formulation	Yes, includes obesity	Yes, ED only
Psychoeducation	Yes, ED and obesity	Yes, ED only
Nutritional counselling	Yes, dietician led	Yes, not dietician led
Behavioural monitoring	Yes, with appetite cues	Yes
Multidisciplinary	Yes	No
Session (number)/duration (months)	30/6	30/6
Weight loss management included	Yes	No
Behavioural activation	Yes	No
‘Healthy’ exercise encouraged	Yes	No
Emotion regulation skills	Yes	Yes

*CBT* cognitive behavioural therapy, *CBT-E* cognitive behavioural therapy-enhanced, *ED* eating disorders, *HAPIFED* Healthy Approach to WeIght management and Food in Eating Disorders

The primary aim of this paper is to detail the protocol (version registered as at 11 July 2015) of a single-blind superiority RCT that compares HAPIFED with CBT-E in weight loss efficacy. A second aim will be to investigate the safety of the introduction of weight loss management particularly for people with BN, but also for those with BED who are overweight/obese.

The primary hypothesis of the RCT will be that at end-of-treatment, 6-month and 12-month follow-up, participants receiving HAPIFED will have lost more body weight than those receiving CBT-E. Secondary hypotheses will be that participants receiving HAPIFED compared to CBT-E group will have significantly improved indicators of weight management e.g., reduced body waist measurement, improved cardiovascular and metabolic status, and that participants in either group who achieve binge eating remission will achieve a greater degree of weight loss.

## Method/Design

### Participants

This study will comprise 100 participants recruited from both community and clinics. Assessments and therapy will be conducted in the Eating Disorders Programme (PROATA) clinic in the Department of Psychiatry of the Federal University of São Paulo (UNIFESP), Brazil. The inclusion criteria are: age ≥ 18 years; either sex; a primary eating disorder diagnosis of BN or BED type according to the *Diagnostic and Statistical Manual of Mental Disorders, fifth edition* (DSM-5) and/or the proposed *International Classification of Diseases, eleventh version* (ICD-11) criteria [2, 3]; and a body mass index (BMI) ≥ 27 and < 40 kg/m^2^. The exclusion criteria are: a current diagnosis of psychosis or bipolar disorder and/or a high level of suicide risk; use of weight loss medication; history of bariatric surgery; clinical conditions that interfere with appetite regulation (e.g., Prader-Willi and Cushing syndromes); and currently receiving psychological treatment for eating disorders.

The present study *a priori* power analysis was based on the estimate of a moderate between group effect size (i.e., 0.6) in the primary outcome of weight loss. To achieve this with power of 0.8 and 1-tailed alpha *p* < 0.05, a minimum of 36 participants per group is required according to Cohen’s tables. Allowing for attrition the sample size is 50 per arm and 10 per therapy group.

### Procedure

Recruitment will take place between June 2015 and December 2016. The recruitment will be through PROATA’s treatment waiting list, printed notices and broadcasting of written and oral media. The first contact will be made by the research team by phone or Email. Those who will be considered eligible for the study will be invited for a first interview at which point informed written consent will be completed, inclusion and exclusion criteria confirmed, and a medical history and physical examination including height and weight (with calibrated scale) undertaken. Medical assessment will accord with international guidelines for weight loss management. Laboratory tests will be circulating fasting lipid profile, fasting glucose matched with insulin, liver function, electrolytes, urea, uric acid and creatinine. A semi-structured interview and self-report questionnaires to determine the eating disorder and co-morbid diagnoses and suicide risk will be conducted. A second appointment will be scheduled for a semi-structured interview that confirms the eating disorder diagnosis and completes a detailed assessment of eating disorder symptoms and behaviours (as detailed in the study timeline on Table 2).

**Table 2:** Timetable of administration of assessment measures

	Baseline	Mid-treatment	End-oftreatment	Follow-up (6 and 12 months)
Demographic questionnaire	✓			
Mini International Neuropsychiatric Interview	✓			
Anthropometry^a^	✓	✓	✓	✓
Eating Disorder Examination	✓		✓	✓
Eating Disorder Examination Questionnaire	✓	✓	✓	✓
Other self-report measures^b^	✓	✓	✓	✓
Adaptive function self-report measures^c^	✓		✓	✓
Physical state biomarkers^d^	✓		✓	
Client satisfaction and success of blinding questions			✓	✓

^a^Waist and hip circumferences, pulse and sitting blood pressure, height and weight for BMI calculation; BMI is also monitored weekly in session

^b^LOCES, BES, DASS21

^c^SF-12: ‘days out of role’

^d^Fasting blood glucose, insulin, lipid profile, liver function test, electrolytes, urea, creatinine and uric acid

*BES* Binge Eating Scale, *BMI* body mass index, *DASS-21* Depression, Anxiety and Stress Scale-21, *LOCES* Loss of Control Over Eating Scale, *SF-12* 12-item Short Form Health Survey

Single blinding will be achieved by not informing participants of any specific details of the therapy content. The information at recruitment that will be provided to participants regarding the therapy is as follows: ‘We have developed an approach that integrates techniques from cognitive behavioural therapy for eating disorders with behavioural strategies for weight loss. One is called HAPIFED (a Healthy Approach to Weight Management and Food in Eating Disorders), and the other is called cognitive behavioural therapy-enhanced … If you agree to participate in this study and have met all the inclusion criteria and if outpatient therapy is suitable for you, you will be randomly allocated (through an online programme) to a group therapy programme that will comprise twice-weekly group over about 1 month … You will have an equal chance (like tossing a coin) of participation in one or other treatment’ (translated from Brazilian Portuguese by author PH). Participants will not be informed if they will be in a HAPIFED or in a CBT-E therapy group at any point, and any treatment materials provided to participant will not be identified as being from either HAPIFED or CBT-E manuals.

Those who will be considered eligible for the study will be randomised in blocks of 20 (2 therapy groups) using an online site (www.sealedenvelope.com). One hundred participants will be allocated over 18 months to 10 groups with 10 participants in each group. Five groups will receive HAPIFED (experimental group) and five will receive CBT-E (control group). An investigator external to the site (PH) will administer the allocation which will be concealed and revealed only to trial therapists and therapy supervisors until the final participant completes follow-up.

### Description of interventions

HAPIFED has 5 stages that comprise 1 individual initial clinical assessment session (session 1) with a psychologist followed by 29 × 90-minute group office-based meetings twice weekly for 4 weeks, then weekly, with 2 psychologists over a total of 6 months. These include four conjoint sessions, two with a dietician and psychologist, one with an occupational therapist, and one psychoeducational session introducing internal hunger and satiety monitoring with a medical doctor. All conjoint sessions are within the first stage of therapy (sessions 2–11). In addition, the occupation therapist will make 2 domiciliary (home) visits (in the 5th and 13th weeks of treatment) and 1 phone call contact (between sessions 21 and 22) to each participant. The aim of these visits is to assess the environment and weight-maintaining habits within the home context. The occupational therapist will formulate a personalised behavioural change programme that will identify opportunities and propose modifications to daily routines to increase physical activity levels and improve relationships with food preparation and consumption. Where severe body image distress has prevented physical activity in front of other people, the occupational therapist will support the participant in physical activity around others and practice techniques discussed with the psychologist to use in relevant situations (e.g., how to cope with situations when feeling stigmatised). The phone call is to check progress since the home visits.

Stage 1 (sessions 2–11) begins with psychoeducation that includes information on why ‘diets’ fail, non-hungry and hungry eating, and reviewing the individual’s eating disorder, weight history, family, and medical history relevant to determining both the likelihood of gaining or losing weight with treatment and obesity-related health risks. Information about eating disorders and an introduction to the CBT formulation of the development of the eating disorder are provided. In addition to disordered eating (bingeing, attempted or actual fasting) and compensatory behaviours (e.g., fasting, vomiting, compulsive exercise), it incorporates weight history and the role of life events and mood intolerance; and, where relevant, interpersonal deficits, low self-esteem and clinical perfectionism. In-session weighing, regular eating and monitoring of key behaviours are introduced. Session 4 includes a presentation on appetite regulation and the rationale for an approach of modifying eating according to internal cues, as outlined in the book *The Don’t Go Hungry Diet* [22]. Specific steps are outlined to help participants lose weight by listening to their appetite, including hunger and satiety scores that are used to monitor and gain awareness of appetite, as well as increasing fruit and vegetable intake and physical activity. This is an alternate to a conventional weight loss programme, which is based on external measures of control (e.g., counting portion size/number) and explicit messages about restriction (e.g., reduce fat, etc.). In session 6 there is a motivational talk from the occupational therapist, and pedometers are provided to promote physical activity. The pedometer will measure the level of activity participants have in their usual routine as well as increase their awareness of their activity levels. After session 19 the pedometers are provided again to check whether they have been able to make improvements in their routine level of activity as well as a reminder of this need. The pedometers will be proposed to be used for 5 days (3 weekdays and 2 weekend days).

Stage 2 (session 12) is a brief ‘reformulation’ time and for personalisation of the formulation. Stage 3 (sessions 13–-19) has an emphasis on behavioural activation and activity monitoring while also addressing the key behaviours and training in specific behavioural skills. Monitoring of key eating and related behaviours (including binge eating, exercise and urge to exercise, other compensations such as vomiting, and body checking) continues with concomitant ratings of mood and appetite, with an additional emphasis on behavioural activation and activity monitoring. Approaches include appetite-focussed CBT techniques. Monitoring is based more on appetite cues than food monitoring. This aims to direct attention away from an excessive focus on type of food [23]. Behavioural experiments are introduced to prevent eating disorder behaviours, establish regular meal patterns and promote self-control. In this stage, nutritional education and counselling and exercise are addressed. The participants will work to make lifestyle changes to establish more healthy eating and physical activity patterns, and food choices which are less than sufficient to meet energy needs, in order to bring about modest, at least 5 %, of previous body weight loss. This is a level known to improve health outcomes [24]. Exercise is addressed using behavioural activation strategies. Stage 4 (sessions 20–27) introduces Socratic questioning and challenging of beliefs and attitudes which reinforce eating disorder behaviours, such as valuing oneself according to one’s weight and shape, and ‘all or nothing’ dichotomous thinking. Problem-solving is also incorporated here. Mood intolerance is addressed with training in specific emotion regulation skills. Behavioural experiments and the appetite-awareness and physical activity plan from phase 2 continue in this phase. Stage 5 (sessions 28–30): as in CBT, the final sessions of HAPIFED involve relapse prevention including reviewing strategies that have successfully helped. By the end of therapy, the goal is to achieve a regular pattern of eating, of varied and appropriately sized food portions, with mild to modest energy restriction. Binge eating and self-induced vomiting and other behaviours are intended to be reduced or absent, and food, eating and weight are envisaged to no longer dominate the patient’s self-view.

Four group follow-up sessions over the subsequent 6 months are also offered to participants. These sessions are conducted by the psychologists who led the group. The aim is to address relapses, review ongoing progress and promote continued improvement.

The comparison intervention is the standard leading evidence-based therapy for BN and BED, CBT-E [25–27]. In this study the broad version of CBT-E [18] with additional mood intolerance and other modules will be used and will match HAPIFED in session duration and number. There will be 1 individual session at the start of treatment plus 29 group sessions over 6-months in this RCT. CBT-E uses therapists from a single discipline, in this study, psychology. CBT for BED and BN has been used successfully in a group format [28, 29].

The structure of HAPIFED has been based on the structure of CBT-E. CBT-E [18] is organised in four stages. Stage 1 (sessions 0–-7) comprises twice-weekly appointments which aim to engage the patient with the treatment, create the formulation, provide information and introduce in-session weighing and regular eating. Stage 2 (sessions 8–9) encompasses the revision of the progress and of the formulation, trying to identify any difficulty to proceed. Stage 3 (sessions 10–17) addresses the mechanisms that maintain the symptomatology and the last stage (sessions 18–20) prepares the patient to continue the progress obtained by the treatment.

### Outcomes

The primary outcome of this intervention will be a reduced body weight sustained to 12 months after completion of the intervention. Secondary outcomes will be (i) improved metabolic indicators of weight management; (ii) reduction in eating disorder symptoms, namely binge eating, loss of control over eating, driven exercise, purging, and self-induced vomiting and dysfunctional cognitions (weight and shape overvaluation and eating concerns); (iii) being less than 1 standard deviation above the community mean for global score on the Eating Disorder Examination (EDE) [18]; (iv) improved adaptive function (‘days out of role’), physical and mental health-related quality of life (HRQoL); and (v) reduced levels of depression and anxiety.

### Assessment

Participants will be assessed 4 times: at baseline, 6 months (end of therapy), 12 months (6 months after end of therapy) and 18 months (1 year after end of therapy). Assessments will be conducted on all participants including those who do not complete treatment.

The timing of administration of assessment measures is shown in Table 3.

1. Measures administered only at baseline:
  1.1. A self-report questionnaire will be used to assess sociodemographic and clinical variables (age, gender, race, occupation, marital status, education, eating disorder onset and lifetime history of eating disorders) and will include a specific questionnaire to determine income status, namely the ‘Critério de Classificação Econômica Brasil 2014’ [30].
  1.2. Mini International Neuropsychiatric Interview (MINI). The MINI is a reliable and valid structured interview for psychiatric diagnoses according to the DSM [2]. Only the MINI-5 [31] for DSM-IV [32] is translated into Portuguese. Thus, the MINI-5 will be used in this study, with modifications to the coding of all disorders to accord with DSM-5 diagnoses as in the MINI-7 [33].
2. Measures repeated from baseline to 12-month follow-up:
  2.1. Anthropometry will include waist and hip circumferences (mean of 3 measures), pulse and sitting blood pressure. Height and weight will be measured with a stadiometer and calibrated scale, from which BMI (kg/m^2^) will be calculated.
  2.2. Eating disorder psychopathology instruments:
    2.2.1. Eating Disorder Examination Edition 17.0D [34]: the EDE is the ‘gold standard’ semi-structured interview used to assess eating disorder symptoms and diagnoses. Four subscales can be derived measuring severity of dietary restraint, eating, shape and weight over concern. These are averaged for a global score. Version 16.0 [18] of the EDE was translated to Brazilian/Portuguese by collaborators from the Federal University of Rio de Janeiro (Silvia Freitas and José Carlos Appolinario), by authors of this paper (MAP, AC) and by a PROATA eating disorder specialist (Christina Morgan – CM), back-translated to English by a certified translator and the final version approved by one of the authors of EDE (O’Connor M). The reliability and concurrent validity of the translated EDE was tested and found to be satisfactory. There was 80 % inter-interviewer agreement (kappa 0.69) on the diagnosis made with the EDE interview and 77.3 % agreement (kappa 0.68) (unpublished data provided by author AC). The diagnosis was made according to the eating disorder module of the Structured Clinical Interview for DSM-IV Axis I Disorders-Patient Edition (SCID-I/P) interview [35] (Portuguese version) [36]. Small adjustments were made by the author MAP, so that the Portuguese version used in the study is now consistent with the 17th version of the EDE [34], in order to derive DSM-5 diagnoses.
    2.2.2. Eating Disorder Examination Questionnaire (EDE-Q) [37]: a self-report measure is derived from the EDE interview. Like the EDE, it has four subscales (dietary restraint, eating, weight and shape concerns). An unpublished Brazilian/Portuguese translation of the more recent 28-item version of the EDE-Q (V. 6.0) [18] was prepared by CM and MAP, and this version will be used. This translation utilised the validated European Portuguese version of the original 36-item EDE-Q [38], which was then modified to be consistent with Brazilian/Portuguese.
    2.2.3. Loss of Control over Eating Scale (LOCES) [39]: a 24-item self-report that examines the presence of loss of control during the binge eating episodes in the last 4 weeks. A Brazilian/Portuguese translation and assessment of psychometric properties (factor analysis and convergent validity) has been completed (da Luz et al., manuscript submitted).
    2.2.4. Binge Eating Scale (BES) [40]: a 16-item self-report instrument that has been translated and validated in a Brazilian sample [41]. This scale evaluates the presence and severity of binge eating. It has adequate psychometric properties in the Brazilian version, with a cut-off point of 17 for the screening of eating disorders in obese individuals seeking treatment for weight loss.
  2.3. General psychopathology instrument:
    2.3.1. Depression, Anxiety and Stress Scale (DASS-21) [42, 43] in a translated and validated Brazilian/Portuguese version [44]. This self-report instrument measures the presence and severity of depressive symptoms, anxiety and stress.
  2.4. Adaptive function:
    2.4.1. Health-related quality of life will be measured with the 12-item Short Form Health Survey (SF-12) [45]. This is a self-report questionnaire that measures health-related quality of life, subdivided in 2 scales – Physical Health Component Summary scales (PCS) and Mental Health Component Summary scales (MCS). A score lower than 30 on the MCS indicates severe impairment in mental health, and a score from 31 to 40 indicates moderate impairment. This is a widely-used measure with sound psychometric properties and has been translated into Brazilian/Portuguese [46].
    2.4.2. Disability will be assessed using a question analogous to the ‘days out of role’ questions employed in the Australian National Survey of Mental Health and Well-Being [47]. Participants will be asked to record the number of days after the following question: *During the past 4 weeks, on how many days, if any, were you unable to complete your work, study or household responsibilities because of any problem with your (physical or emotional) health?* This was translated by the author MAP.
3. Measures only at baseline and end-of-treatment.
  The following medical pathology tests will be performed at baseline and end-of-treatment only: fasting circulating glucose, insulin, lipid profile, liver function test, electrolytes, urea, creatinine and uric acid.
4. Measures at end-of-treatment only.
  4.1. Client/patient satisfaction questionnaire [48]. This eight-item scale assesses general satisfaction with services. It has a high internal consistency (*α* = 0.93) and correlates with therapist’s assessment of client satisfaction and with early treatment drop out. This was translated by the author MAP.
  4.2. Participants will be asked which group, experimental or control, they thought they were in. When all participants have completed 12-month follow-up and data has been processed, participants will be debriefed, and full information will be provided about HAPIFED and CBT-E.

**Table 3:** Time schedule for the randomised controlled trial

Semesters	2015 Jan/Jun	2015 Jul/Dec	2016 Jan/Jun	2016 Jul/Dec	2017 Jan/Jun	2017 Jul/Dec	2018 Jan/Jun	2018 Jul/Dec
Stages								
HAPIFED and CBT-E groups 1 and 2 Recruitment and intervention	x	x						
HAPIFED and CBT-E groups 3 and 4 Recruitment and intervention		x	x					
HAPIFED and CBT-E groups 5 and 6 Recruitment and intervention			x	x				
HAPIFED and CBT-E groups 7 and 8 Recruitment and intervention			x	x				
HAPIFED and CBT-E groups 9 and 10 Recruitment and intervention				x	x			
HAPIFED and CBT-E groups 1 and 2 Follow-up (6 and 12 months)			x	x				
HAPIFED and CBT-E groups 3 and 4 Follow-up (6 and 12 months)				x	x			
HAPIFED and CBT-E groups 5 and 6 Follow-up (6 and 12 months)					x	x		
HAPIFED and CBT-E groups 7 and 8 Follow-up (6 and 12 months)					x	x		
HAPIFED and CBT-E groups 9 and 10 Follow-up (6 and 12 months)						x	x	
Development and maintenance of the database		x	x	x	x	x	x	
Preparation and publication of results					x	x	x	x

*CBT-E* cognitive behavioural therapy–enhanced, *HAPIFED* Healthy APproach to WeIght management and Food in Eating Disorders

### Treatment fidelity

Participants will be assigned to therapists who conduct both forms of treatment, to control for potential non-specific therapist effects. An occupational therapist will conduct two home-based sessions. In preparation for this RCT, therapists experienced with CBT-E and HAPIFED (PH, JS) have trained all therapists (psychologists, occupational therapist and dietitian) and conducted monthly telephone supervision sessions for 2 pilot therapy groups, one for CBT-E and one for HAPIFED (August 2014 to February 2015; da Luz et al., manuscript in preparation). In addition, an onsite Brazilian therapist CM has undergone the Oxford CBT-E online training. De-identified audio recordings were made of all pilot sessions to assist with training. Supervision of therapists during the trial will be provided by a meeting each month with PH, with additional specialist supervision by AS with expertise in appetite regulation and other specific approaches to weight management. Both therapies will be conducted in accordance with the treatment manuals. PH will visit Brazil twice a year for 3 years for face-to-face meetings with the Brazilian team. A random 10 % sample of de-identified digitally recorded audiotaped sessions will be reviewed by AC for fidelity.

### Data collection, management and analysis

Researchers conducting outcome data entry will be blind to the study group of participants. Between-group outcome analyses will not begin until after the final 1-year follow-up. Governance of data and an independent Data Monitoring Committee (DMC) responsible for interim mid-point in recruitment safety and futility analyses of de-identified data will be conducted at the University of Western Sydney site. The DMC will be composed of a biostatistician with expertise in RCTs, and two senior independent clinical researchers with expertise in RCTs. The trial will be discontinued if assessed to be futile and/or participants’ binge eating is increasing significantly and/or attrition is over 50 %. Therapists under supervision who are not blind to treatment arm will be responsible for the safety of individual participants and will receive directly any concerns about an individual from the DMC.

STATA (StataCorp, College Station, TX, USA) for most statistics will be used. Baseline univariate between-group tests will be done to compare groups on outcome variables, clinical and demographic data. Data will be analysed following ‘intention-to-treat’ principles. Generalised estimating equations [49] with a logit response function will be used for dichotomous outcomes, such as achieving a 5 % reduction in body weight. Linear mixed effects modelling [50] will be used to test for between-group differences in the continuous outcome measures, namely weight loss, levels of ED symptom change, adaptive function, quality of life, depression and anxiety levels, waist circumference, blood pressure, and biochemical markers such as circulating lipid or glucose levels. Putative covariates will be diagnostic group (BN or BED) and use of psychotropic medication. Predictors of outcomes at 6-month and 12-month follow-up to be tested will include the rate of early (by mid-treatment) reduction in binge eating.

### Ethics and dissemination

The study has been approved by the Human Research Ethics Committee of the Federal University of São Paulo, Brazil (CAAE 43874315.4.0000.5505). Written informed consent including information on confidentiality provisions will be obtained from each subject. Only investigators and authorised research personnel will have access to the database. Reports will be made regularly to the committee including results of interim safety analyses by the DMC.

The trial results will be submitted to a peer-reviewed scientific paper, and authors will present results at appropriate scientific meetings and community and advocacy organisations, as well as being released to the media following publication.

The Standard Protocol Items: Recommendations for Interventional Trials (SPIRIT) Checklist for recommended items to address in a clinical trial protocol is found as an additional file (see Additional file 1).

## Discussion

HAPIFED adopts a new approach that integrates the most effective treatment for BN and BED (CBT-E) with BWLT strategies that are most likely to lead to longer-term weight loss [51]. It is the first such therapy proposed that recognises that management of the person who is obese and has an eating disorder needs to be of increased intensity and duration if improved weight management and sustained behavioural change is to be achieved, similar to the intensity used in the underweight eating disorder patient [18].

The currently proposed study is a RCT with allocation concealment. It is limited in that only blinding of participants (but not therapists) will be attempted. Single blinding is being attempted by participants being informed only that they will be randomised to either CBT-E or HAPIFED group-based therapy of equal session number and duration. They will not be informed of any specific details of the therapy content. Whilst it is possible that patients familiar with psychological therapies would correctly guess what type of therapy group they are in, this may be less likely in Brazil where English literacy is not common and to our knowledge the CBT-E manual has not been published in Portuguese. We will also check for the success of the participant blinding.

The choice of control therapy for HAPIFED also has some limitations. It was made on the basis that whilst BWLT had been found to be efficacious and safe in BED [26] longer-term outcomes compares less well to CBT [27] and it is not known to be effective or safe in BN [26]. Therefore, CBT-E, the lead evidence-based therapy for BN, an evidence-based therapy for BED and recommended by international treatment guidelines, was chosen as our control therapy. CBT-E is, however, an active therapy akin to ‘standard care’ that controls for non-specific effects of therapy and also for specific effects on reducing eating disorder symptoms, but it is not a weight loss therapy. Thus, the study is not able to determine the efficacy of weight loss in HAPIFED compared to other weight loss treatments, but only compared to a control condition without an active weight loss component.

The expected outcomes and significance of this proposed trial are that HAPIFED has the potential to improve health outcomes for the rapidly increasing number of people with obesity and eating disorders, and to help close the treatment gap for adults with co-morbid eating and weight disorders. Effective approaches to improve weight management for this group will reduce the well-known personal and community burden of obesity, as well as the impact on health systems from physical (such as high blood pressure) and mental health (such as depression [52]) consequences of obesity. We anticipate that this study will also inform the sample size required for future trials to show whether HAPIFED can induce clinically significant weight loss while also producing as great a benefit as CBT-E for the reduction of eating disorder symptoms.

## Trial status

Recruitment, assessment and the first therapy groups have commenced.

## Additional file

Additional file 1:
**SPIRIT_Fillable-checklist – Palavras et al.** (PDF 171 kb)

## Abbreviations

BED: binge eating disorder
BES: Binge Eating Scale
BMI: body mass index
BN: bulimia nervosa
BWLT: behavioural weight loss treatment
CBT: cognitive behavioural therapy
CBT-E: cognitive behavioural therapy-enhanced
DASS-21: Depression, Anxiety and Stress Scale
DMC: Data Monitoring Committee
DSM: *Diagnostic and Statistical Manual of Mental Disorders*
EDE: Eating Disorder Examination
EDE-Q: Eating Disorder Examination Questionnaire
HAPIFED: Health APproach to eIght management and Food in Eating Disorders
HRQoL: health-related quality of life
ICD: *International Classification of Diseases*
LOCES: Loss of Control over Eating Scale
MCS: Mental Health Component Summary scales
MINI: Mini International Neuropsychiatric Interview
PCS: Physical Health Component Summary scales
PROATA: Eating Disordes Programme
RCT: randomised controlled trial
SCID-IP: Structured Clinical Interview for DSM-IV Axis I Disorders-Patient Edition
SF-12: 12-item Short Form Health Survey
SPIRIT: Standard Protocol Items: Recommendations for Interventional Trials
UNIFESP: Federal University of São Paulo
